# Nitric Oxide: The Coming of the Second Messenger*

**DOI:** 10.5041/RMMJ.10038

**Published:** 2011-04-30

**Authors:** Ferid Murad

**Affiliations:** Nobel Laureate in Medicine, 1998; Professor and Director Emeritus, Texas Nobel Scholar, The University of Texas, Houston, TX, USA

## INTRODUCTION: INTRACELLULAR COMMUNICATION

The concept of communications between cells or cell signaling dates back over 100 years to Pavlov. He discovered that neuronal signals, first generated by the smell of food and later by the ringing of a bell, enhanced gastric secretion. The neurons communicated with cells in the stomach.

Today it is well established that cell signaling is a universal phenomenon, occurring throughout the body and even between unicellular organisms such as yeast, fungi, and bacteria. The molecules that are used for the purpose of communicating between cells are diverse and comprise amino acids, peptides, proteins, and other organic molecules. These molecules, which number in the hundreds, were initially called “first messengers” and are now called hormones, cytokines, growth factors, paracrine substances, neurotransmitters, and a variety of other names. These molecules find their target cell by identifying and binding to a receptor that is mostly located on the surface of the target cell. This binding ensures the specificity of the interaction, since only cells with specific receptors will bind to specific ligands. The binding of the ligand to the receptor initiates a biochemical cascade, resulting in the accumulation of an intracellular second messenger, which then goes on to trigger the desired effect on the cell.

The first second messenger, which was discovered in 1957, was cyclic adenosine monophosphate, or cAMP. Others came along in the ensuing 10–15 years. Today, we know there are many such molecules, including cyclic guanosine monophosphate (cGMP), nitric oxide (NO), calcium, diacylglycerol, phosphatidylinositols, and more, some surely yet to be discovered. Many of these discoveries eventually led to a Nobel Prize.

## THE MOLECULAR VERSATILITY OF NITRIC OXIDE

NO is a unique messenger. It is a gas, although it does dissolve in an aqueous solution. It is a free radical with an unshared electron, which will react with thiol groups on proteins to form new complexes. In contrast to other second messengers, NO does not require energy to transport itself in and out the cell. It can go where it wants, but it does not travel far because it is a reactive free radical. NO functions within the cell that produces it as a second messenger. It functions as a paracrine molecule able to travel 10–20 cell diameters to regulate the biology in adjacent cells. It can be complexed to glutathione, hemoglobin and other proteins, and disassociate from these complexes, thus enabling NO to travel in the bloodstream and affect cells at a distance. This qualifies NO as a hormone, a paracrine substance and a second messenger. No other known substance fulfills such diverse roles.

The use of NO as a drug began in the later part of the nineteenth century when nitroglycerin was first discovered to treat angina pectoris in the 1870s. The mechanism of action would not be elucidated until close to a century later. Some of NO’s sources and effects are summarized in Table 1.

## THE LONG ROAD TO DISCOVERY

The discovery of NO as a second messenger began with the discovery of cyclic GMP as an organic molecule. Southerland, Rall, Fisher and others showed that the target for cyclic adenosine monophosphate (cAMP) was a protein kinase whose function was to phosphorylate specific proteins downstream, thereby causing a specific function in the cell. The first such kinase discovered was phosphorylase kinase. Today, we know that there are hundreds of such kinases, and many second messengers work by selectively activating a specific protein kinase. When the target protein is phosphorylated, it causes a conformational change to that protein. If that protein is an enzyme, phosphorylation will change the activity of that enzyme. If you phosphorylate a structural protein, the shape, structure, function of that protein can change. The effects of these alterations can be smooth muscle relaxation or contraction, changes in motility, etc. The concept of signaling is quite simple, but, due to the hundreds of potential protein substrates that the messenger can affect, the specific pathway can become confusing. There are, for instance, 10 or 11 adenylyl cyclases, 7 or 8 guanylyl cyclases, 10 or 11 phosphodiesterase kinases, and a myriad of protein kinases, all potential protein substrates in specific pathways.

During the 1960s, many big labs were working on elucidating the roles of cAMP, so I decided to go to a less crowded area and work on cyclic guanosine monophosphate (cGMP). I sought to answer two questions: 1) I wanted to know precisely how hormones and various first messengers or ligands regulated the activity of guanylate cyclase. By elucidating the workings of this molecular coupling, we could intervene and increase or block the binding of the first messenger to its ligand. This could potentially lead to drug discoveries for treating various endocrine disorders. 2) What role did cGMP play? At the time we had no clues as to its biological function or functions.

We chose to work with guanylate cyclase, and were very surprised by the results of our first experiments. We made homogenates from liver, heart, and other tissues and found that there were two activities: one in the high-speed supernatant (soluble) fraction, and the other in the high-speed particulate fraction. The kinetic properties and behavior of these activities were different. We thought we were dealing with isoforms, and, if we had two different isoforms in two different compartments, perhaps there were two different pools of cGMP with different functions that respond to different hormones. Proving this would take many years.

We also knew that certain hormones were capable of elevating cGMP levels in intact tissues such as heart and blood vessels but no such an effect was seen in cell-free systems. To fully understand hormone–cyclase coupling, it was imperative to have a cell-free system. With such a system one could add individual components and see the effect on the system.

Having experience with working on cAMP as a student, I decided to add azide to different tissues and found that the effect was tissue-specific and that it only activated the soluble enzyme and not the particulate. In addition, this reaction required oxygen, and there was also a time-lag of several minutes before the rate of the reaction became maximal. We were able to enhance the effect with thiols such as cystine. All this suggested that the effects of azide, hydroxylamine, and of nitrite were indirect. They were not proximal activators of guanylyl cyclase but were probably being converted to something else or influencing some other molecule. We also noticed that when azide was added to cell cultures or liver and brain slices, the levels of cGMP would rise. That enabled us to use azides instead of hormones to achieve a similar effect.

We took two crude supernatant fractions: a liver fraction that was activated by azide (azide responsive) and a cerebral cortex fraction that was not activated by azide (azide non-responsive). We also had a non-responsive heart fraction. When we mixed liver and heart supernatants, the azide effect disappeared. We assumed that the reason was that the heart tissue possessed an inhibitor. That was a simple but very important experiment. We later went on to purify the inhibitor which turned out to be the proteins hemoglobin and myoglobin.

When we mixed liver with cerebral cortex, the azide effect was potentiated. We therefore assumed the existence of an activator in the liver tissue that is absent in the cerebral cortex. We discovered that the activating factor t was a catalase. The inhibitors, hemoglobin and myoglobin and the activator, catalase are all heme proteins. The fact that NO was both inhibited and activated by heme proteins was intriguing, but at the time we did not know the significance of this discovery.

At the time, we were working on an almost 90% pure smooth muscle preparation. We found that azide elevated cGMP levels in smooth muscle and caused muscle relaxation. Dose–response curves, time-courses, and other tests convinced us that one of the effects of cGMP was smooth muscle relaxation. We initially avoided using vascular smooth muscle because it is so heterogeneous, being only about 30% smooth muscle. We later found that cyclic GMP induced relaxation in vascular smooth muscle as well.

Using my medical background, I contemplated whether other smooth muscle relaxants such as nitroglycerine and nitroprusside had similar effects on cGMP levels and indeed they did. We ended up with a long category of compounds we called nitro-vasodilators, although not all were nitro compounds. The list included azide, hydroxylamine, nitrite, phenylhydrazine, nitroprusside, nitrosamines, and others. We assumed that all those compounds were converted to an intermediate. We postulated that the activator must be nitric oxide because we knew that hemoglobin and myoglobin blocked the effects of all these activators, and we knew from the literature back in the 1940s that nitric oxide had high affinity for the heme prosthetic group in hemoglobin and myoglobin. We synthesized nitric oxide in the fume hood, combined sodium nitrite, sulphuric acid, and ferrous sulphate with a stir bar and vented the gas into our cyclase incubations. The nitric oxide activated every preparation we tested. Nitric oxide was indeed the intermediate that we were looking for.

The fact that we had an activator that is also a free radical was a radical idea. This idea led to a thought that other enzyme systems might be regulated by free radicals as well.

## NITRIC OXIDE – MECHANISMS OF ACTION

Purified homogeneous guanylyl cyclase is activated 200–400-fold, a huge increase of Vmax, with as little as nanomolar concentrations of nitric oxide. This is what a nitro-vasodilator does in smooth muscle. It quickly elevates cGMP, causing relaxation, whereby the messenger goes away, but relaxation persists. The reason for this is that cGMP has a short half-life; it is degraded and released from the cell. The physiologic effect downstream persists because the cyclic GMP-activated protein kinases and the phosphorylated proteins have a longer half-life and a slower turn-over.

Nitroglycerine had been used for a hundred years, and no one understood how it worked. With this discovery, we ascertained that nitroglycerine works as an NO donor to increase cGMP levels and cause smooth muscle relaxation.

The question then arose, if NO affects the tissue as an exogenous agent, is it mimicking an endogenous pathway? Could nitric oxide be an organic intracellular messenger? It had been unheard of that a free radical is a messenger molecule that regulates the actions of hormones. The technology of measuring concentrations of nitric oxide and its oxidation products, nitrite and nitrate had not yet been developed because the concentrations of NO that are required in these biological systems are incredibly low in the micro- and nano-molar range,. It took another 7 to 8 years to develop this technology.

Around that time, in 1980, Robert Furchgott was able to show for the first time that a group of drugs that were known to be hypotensive in animals and man, such as acetylcholine, histamine and bradykinin,, caused relaxation of blood vessels in an organ bath in the laboratory. Previous investigators had failed to see such an effect *in vitro.* Furchgott showed that if he made his vessel preparations in rings instead of in longitudinal segments, the drugs worked. The rings preserved the integrity of the endothelium, whereas longitudinal cuts adversely affected the endothelium.

The intact endothelium produced a substance that Furchgott termed the endothelium-derived relaxing factor (EDRF). This factor had a very short half-life of only a few seconds. I later showed that this endothelial dependent vasodilator worked through elevating levels of cGMP. The increase in cyclic GMP takes place in the smooth muscle compartment. If the endothelium is damaged, there is no cGMP increase, no relaxation, and no effect from these endothelial vasodilators. Furchgott and my experiments were the spark that was needed to excite the research community about cGMP. We finally had a physiological function, namely, smooth muscle relaxation.

Both NO and EDRF raised cGMP levels, which caused phosphorylation of proteins downstream. With the help of 2D gels, we were able to identify some of those proteins.

**Figure 1:**
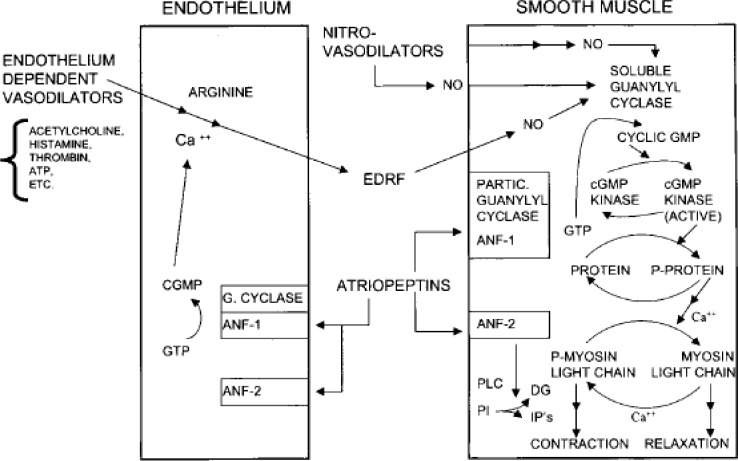
Effects of endothelium-dependent vasodilators, nitro-vasodilators, and atriopeptins in rat aorta segments. ANF-1,2, atrial natriuretic factor 1 and 2; DG, diacylglycerol; EDRF, endothelium-derived relaxing factor; IPs, inositol phosphates; NO, nitric oxide; PI, phosphoinositides; PLC, phospholipase C. (From: Murad F. Cyclic guanosine monophosphate as a mediator of vasodilation. J Clin Invest 1986;78:1–5. With permission.)

Figure 1 depicts a blood vessel with its endothelial lining, the underlying smooth muscle compartment, and the three categories of vasodilators: nitro-vasodilators, endothelium-dependent vasodilators, and atriopeptins. They all function by increasing cyclic GMP production.

The members of the first category, the nitro-vasodilators, release NO either spontaneously or enzymatically, depending on their structure, oxygen tension, and the pH of the blood. Nitroglycerine, for example, requires an enzyme to remove the nitro and make NO. The NO then binds to a heme prosthetic group on soluble guanylyl cyclase. When NO binds to the heme group it induces a conformational change on the heme ligand. This lifts the constraint that the heme group placed upon the enzyme, thus activating the catalytic domain of guanylyl cyclase 200–400-fold, resulting in an increase in cGMP levels.

Cyclic GMP activates a protein kinase which phosphorylates a variety of proteins, thus inducing relaxation. Cyclic GMP does this in several different ways. It regulates cytosolic calcium levels by regulating gated ion channels. It also affects the distribution of calcium ions in different intracellular pools. The net effect of cGMP is dephosphorylation of myosin which causes the myosin filaments to slide apart thus relaxing the muscle.

The members of the second category, the endothelial-dependent vasodilators relax the muscles as well, but they require the integrity of the endothelium which is where the receptors are located. The endothelial-dependent vasodilators have no receptors on the smooth muscle cell. Therefore, when the endothelium is damaged, they do not function.

In addition, the endothelial-dependent vasodilators increase calcium in endothelial cells to increase the production of EDRF, known today as nitric oxide. NO then goes over to the smooth muscle, and the subsequent cascading is identical. Since these agents need an intact endothelium, they are not used in intensive care units or intensive coronary care units since many patients in those units have a dysfunctional endothelium.

The members of the third category are the atriopeptins. DeBol purified and sequenced a peptide that was found in the granules of the cardiac atria. These peptides were dubbed atrial natriuretic factors by deBol. The atrial natriuretic factor is a vasodilator and also a natriuretic agent in the kidney. These peptides are similar to *Escherichia coli’s* enterotoxin that causes diarrhea. *E. coli’s* heat-stable enterotoxin activates a particular guanylate cyclase to increase cyclic GMP in the intestinal mucosa. That, in turn, phosphorylates the cystic fibrosis trans-membrane conduction regulator to secrete chloride, sodium and water to cause diarrhea. The atriopeptins have a guanylyl cyclase receptor. The particulate cyclases are peptide receptor cyclases. There are about six of these particulate isoforms: GCA is the receptor for atriopeptins, as is GCB. The enterotoxin receptor is GCC. It is only found in the intestinal mucosa and in colon cancer, and has become a biomarker for colon cancer.

## NITRIC OXIDE-PRODUCING PATHWAYS: ENZYMES AND CO-FACTORS

Having identified many vasodilators that use NO, it became apparent that there had to be some enzymes making nitric oxide. In the mid to late 1980s, several laboratories set out to describe these enzymes. They discovered three isoforms of nitric oxide synthase (NOS) which they initially called neuronal NOS, inducible NOS (because it was induced by proinflammatory cytokines), and endothelial NOS (Table 2). Because all three isoforms are ubiquitous, we suggested to call them NOS-1, 2, and 3, according to the chronological sequence in which they were first purified and cloned.

**Figure 2:**
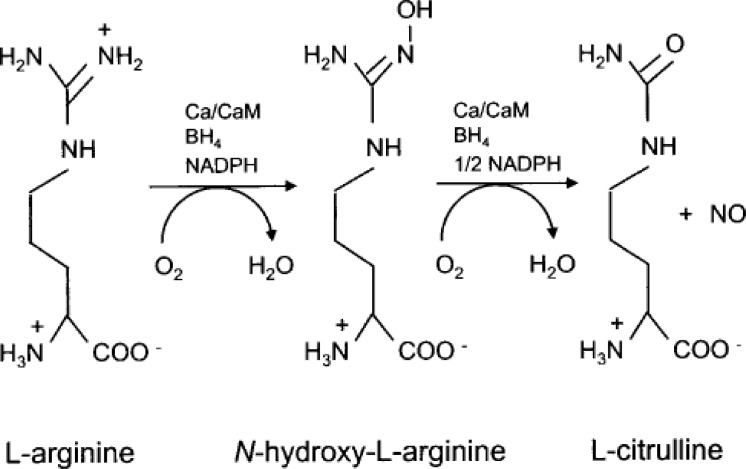
The nitric oxide synthetic pathway.

All three enzymes are heme proteins. Their genes reside in three different chromosomes. They share a 50%–60% homology, and are also homologous with cytochrome P-450. They are very complicated enzymes. They require an array of co-factors such as oxygen, NAPH, and arginine. They are calcium/calmodulin-dependent enzymes (less so for NOS-2 because it comes off the ribosome with calmodulin already bound to it) and are post-translationally regulated by phosphorylation, myristoylation, and palmitation. These enzymes oxidize the terminal guanidino nitrogen of L-arginine to an intermediate hydroxy-arginine, and further oxidize it to form nitric oxide and citrulline (Figure 2). NOS comes off in a very active, high-output state so that when it is activated, it produces substantial amounts of NO. NOS also participates in many inflammatory processes.

Another important NOS co-factor is tetrahydrobiopterin. If tetrahydrobiopterin is oxidized to dihydrobiopterin, the enzyme becomes uncoupled and NO is no longer produced. Instead, the enzyme makes superoxide anions which are detrimental because they are an incredible trap for nitric oxide. Tetrahydrobiopterin reacts in an almost diffusion-limited way to make peroxynitrite, a new, more reactive species which will phosphorylate tyrosinyl residues in proteins and lipids.

**Figure 3:**
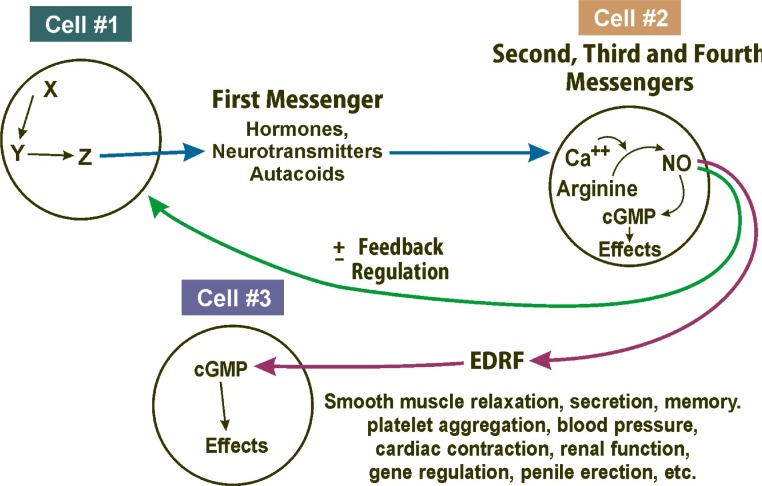
The nitric oxide (NO) and cyclic GMP (cGMP) cell signalling pathway (cells talking to each other). EDRF, endothelium-derived relaxing factor.

The classic NO pathway is as follows (Figure 3): A hormone (or first messenger or ligand) binds to its appropriate receptor, which then often regulates a co-factor for nitric oxide synthase. It frequently regulates calcium, but can regulate some of the other co-factors as well. The nitric oxide synthase converts arginine to EDRF (which is in fact nitric oxide). NO activates soluble guanylyl cyclase to make cyclic GMP. Alternatively, NO can interact with cyclic GMP to activate a protein kinase. The kinase will then phosphorylate other specific proteins to cause an effect.

The different points in the pathway constitute molecular targets for drug development. For instance, hormone agonists and antagonists can be created and co-factor can be modified. There are molecules that will inhibit nitric oxide synthase, like arginine analogs, or guanidine compounds. There are compounds that will scavenge the NO in the bloodstream and are therefore used for people with septic shock or hypotension during renal dialysis. There are a number of candidate compounds whose purpose is to potentiate the activation of guanylyl cyclase by NO.

Inducing the production of cGMP by NO or other pathways is overall beneficial for the body. It enhances blood-flow to tissues by being vasodilators. In addition, it inhibits platelet aggregation in blood clotting and prevents the capping and rupturing of atherosclerotic plaques which can go downstream and cause infarctions.

Another important effect of nitric oxide is the formation of nitrosothiol groups in various proteins. More than 100 known proteins are nitrosated on their cystine residues. This modification may be significant, since some of the nitrosated proteins such as the MMDA receptor, cascade inducers, and transcription factors are receptors and might represent other signaling pathways.

A very important pathway is the interaction of nitric oxide with the superoxide anion to form peroxynitrite. What we have learned from work in our labs and from other labs is the evidence of peroxynitrite formation by protein tyrosine nitration. Indeed, the association of peroxynitrite association with an increase in NOS-2 levels has evolved as a biomarker of inflammatory diseases like Alzheimer’s disease, Parkinson’s disease, atherosclerosis, myocarditis, nephritis, colitis, and arthritis. One avenue of research is to prevent the occurrence of adverse NO reactions.

Because competing pathways exist, nitric oxide can also go off onto other tracks. NO can be oxidized to nitrite/nitrate, which were thought to be dead-end metabolites of no effect. It turns out that the nitrite in processed meats such as hot dogs and sausages result in the production of nitric oxide in the body. The salivary glands in the mouth accumulate nitrates. Saliva is secreted into the oral cavity. The bacteria in the mouth reduce the nitrates to nitrite. The nitrite is swallowed and the stomach acid converts it to nitric oxide. In this way, the salivary glands are “talking” to the stomach, a connection that Pavlov missed. Therefore, if you do not brush your teeth and do not use mouthwash, the levels of NO, which is cardio-protective, will increase. You will have bad breath and rotten teeth and no social life, but you will have a strong heart, maybe.

## NITRIC OXIDE-BASED DISORDERS AND THERAPIES

Endothelial dysfunction is a disorder that has been described for some years now (Table 3). It is a disorder in which the blood vessels do not make enough nitric oxide. Patients with hypertension, diabetes, atherosclerosis, smokers, and perhaps obese people as well, do not make enough nitric oxide in their blood vessels for a variety of reasons. All these diseases are associated with reactive oxygen species and oxidative stress. I hypothesize that in these conditions, the oxidizing co-factors that are required to make nitric oxide are depleted, whereby superoxide is produced instead of NO. The superoxide reacts with the existing levels of NO, further depleting the NO levels. This vicious cycle continues, resulting in a vascular disease.

Furthermore, all the patients with endothelial dysfunction have increased levels of an arginine metabolite, which is a competitive inhibitor of nitric oxide synthase. Methylated arginine on both guanidino nitrogens, called symmetric dimethylarginine, is a benign molecule. But an asymmetric dimethylarginine, which consists of two methyls on the same nitrogen, is a very important competitive inhibitor of nitric oxide synthase. All the aforementioned blood vessel diseases are associated with elevated blood levels of asymmetric dimethylarginine. Does this mean that arginine supplementation will improve nitric oxide production? Certain animal studies look encouraging although the clinical study results are mixed. The present trend of arginine and antioxidant supplementation is beginning to fit with the biochemistry.

We are quite certain that NO is a neurotransmitter. If you make a NOS-1 knock-out mouse, it will induce small strokes or infarcts in the mouse. If you make a NOS-3 knock-out mouse, the infarcts will be larger. If you do a double NOS-1 and NOS-3 knockout mouse, it will perform very poorly in memory-based tests. It thus appears that NO participates in cerebral function, but we do not know the details.

We know that nitric oxide plays a role in fluid turn-over in glaucoma. Excess nitric oxide in the retina leads to retinal ganglial cell death. There are some thoughts of devising agents that will inhibit NOS in the retina.

Pulmonary hypertension is an additional area where the effects of NO are readily seen. *In utero*, the baby does not need to perfuse its lungs as it gets its oxygen from the mother’s blood via the placenta. Normally, in a mature new-born infant, the blood perfusion of the lungs will open up as the lungs unfold. In contrast, a premature baby maintains its fetal circuit. Due to the lack of surfactant, the lungs cannot unfold, and the pulmonary vessels remain constricted. They shunt blood from right to left through a patent foramen ovale or a patent ductus arteriosus. These are “blue babies”. If these babies are given low concentrations of nitric oxide through a nasal catheter, their pulmonary vessels dilate, the right to left shunt diminishes, and their oxygen concentration and saturation improves. NO is a welcomed therapeutic addition in neonatal units.

I believe that the most known function of NO is in penile erection. We used to have a joke in our lab in the 1970s about filling condoms with NO donors and other goodies. It was a joke but I didn’t realize that it would become so important.

There are many other known processes and diseases in which NO participates. A summary of these can be seen in Table 4.

## FUTURE DISCOVERIES AND USES OF NITRIC OXIDE

We have learnt from microarray studies that nitric oxide regulates many genes. There are many gene messengers that go up, and many that go down. We do not know what it means or how NO is doing it. These processes appear to be cGMP-independent, so it is not a kinase mechanism but something else. Are there transcription factors or co-activators that are influenced by nitric oxide? I do not know.

We are also doing some interesting things with stem cells. Having started with mouse embryonic stem cells about 5–6 years ago, we are presently spending more and more time with human embryonic stem cells and are able to influence the differentiation of embryonic stem cells toward becoming lineages as myocardial cells or neuronal cells by manipulating nitric oxide and cyclic GMP pharmacologically. Our goal is to see if we can influence differentiation in the laboratory in a way that will, at some point, enable us to manufacture tissues for transplantation.

Many diverse roles for nitrous oxide have been discovered in the previous decades. We have tantalizing evidence that NO is important in many additional biological systems. The final chapters on NO’s roles in the body have yet to be started.

## Figures and Tables

**Table 1:** Several sources of nitric oxide and some of its effects.

Sources

Natural substance in the body
Drugs
Car exhaust
Cigarette smoke
Fire

Effects

Ozone depletion and earth warming
Free radical interactions
Numerous biological effects (both beneficial and deleterious):
Angina
Anti-bacterial
Arthritis
Blood pressure
Diabetes
Esophagitis
Gene regulation
Heart contraction
Inflammation
Memory

**Table 2:** Nitric oxide synthesis isoforms.

NOS-1(155kD)	Neuronal, brain, Type I-NOS; central and peripheral neurons, non-adrenergic and non-cholinergic neurons, islets, endometrium, skeletal muscle, etc.
NOS-2(125kD)	Inducible, Type II-NOS; macrophage, liver, smooth muscle, endothelium, heart, etc.; effects of lipopolysaccharides, cytokines, and glucocorticoids
NOS-3(135kD)	Endothelial, Type III-NOS; endothelium, brain, heart, etc.; acylation, phosphorylation

**Table 3:** Endothelial dysfunction (diabetes, hypertension, atherosclerosis, tobacco use).

Elevated asymmetric dimethyl arginine (ADMA)
Elevated reactive oxygen species (ROS)
Decreased NOS co-factors
Decreased NOS activity
Decreased NO production
Increased peroxynitrite formation with increased removal of NO
Possible role of L-arginine and anti-oxidant supplements

**Table 4:** Processes and diseases with NO Participation.

Neurotransmission, memory, stroke
Glaucoma and neural degeneration
Vasodilation, blood pressure, blood-flow
Pulmonary hypertension
Penile erection
Angiogenesis, wound healing
Atherogenesis
Inflammation, arthritis, nephritis, colitis, etc.
Cytotoxicity tissues, pathogens, tumors
Asthma
Tissue transplantation
Septic shock, dialytic hypotension
Platelet aggregation
Gastrointestinal motility
Hormone secretion
Gene regulation
Hemoglobin delivery of oxygen
Stem cell proliferation and differentiation
Bronchodilation

## Abbreviations:

ANF-1,2: atrial natriuretic factor 1 and 2
cAMP: cyclic adenosine monophosphate
cGMP: cyclic guanosine monophosphate
DG: diacylglycerol
EDRF: endothelium-derived relaxing factor
IPs: inositol phosphates
NO: nitric oxide
NOS: nitric oxide synthase
PI: phosphoinositides
PLC: phospholipase C

